# Concentration of Essential, Toxic, and Rare Earth Elements in Ready-to-Eat Baby Purees from the Spanish Market

**DOI:** 10.3390/nu15143251

**Published:** 2023-07-22

**Authors:** Luis Alberto Henríquez-Hernández, Andrea Carolina Acosta-Dacal, Luis D. Boada, Manuel Zumbado, Lluis Serra-Majem, Octavio P. Luzardo

**Affiliations:** 1 Toxicology Unit, Research Institute of Biomedical and Health Sciences (IUIBS), Universidad de Las Palmas de Gran Canaria, Paseo Blas Cabrera Felipe s/n, 35016 Las Palmas, Spain; andrea.acosta@ulpgc.es (A.C.A.-D.); luis.boada@ulpgc.es (L.D.B.); manuel.zumbado@ulpgc.es (M.Z.); octavio.perez@ulpgc.es (O.P.L.); 2 Spanish Bimedical Research Centre in Physiopathology of Obesity and Nutrition (CIBERObn), Paseo Blas Cabrera Felipe s/n, 35016 Las Palmas, Spain

**Keywords:** risk assessment, baby food, chemical elements, heavy metals, rare earth elements, food safety

## Abstract

Background: The infant population is particularly sensitive, so the risk posed by their diet must be analyzed. The aims of the present study were (i) to determine the contents of 38 elements in 159 samples of ready-to-eat baby food sold in Spain and (ii) to estimate the dietary intakes and risk assessments of these elements in name brands and store brands in infants ranging between 6 and 12 months of age. Methods: A list of essential, non-essential/toxic elements, rare earth elements (REEs), and other hi-tech-related elements that are currently considered as emerging environmental pollutants were measured in ready-to-eat baby foods by ICP-MS. Results: Fish purees showed the highest concentrations of mercury (28.1 ng/g) and arsenic (346.2 ng/g). The levels of manganese, molybdenum, and chromium exceed the adequate intake, being higher in the case of store brands. The acute hazard index was above 1 for molybdenum and manganese. A risky consumption of thallium and mercury was observed, being higher among name brands. The risk associated with the consumption of REEs was low, although its presence should be highlighted. Conclusions: This is the first time that these chemical elements have been measured in ready-to-eat purees for babies. The presence of some of them, such as mercury, should be sufficient to monitor the levels of these contaminants in food intended for such a sensitive population as children.

## 1. Introduction

Feeding during the first year of life is fundamental to children’s growth and development. According to World Health Organization (WHO) recommendations, breast milk should be the only food for the first six months of life [1]. According to data from the Spanish National Institute of Statistics, 24.7% of babies were exclusively breastfed for 6 months, whereas 14.0% were mixed breastfed [2]. From then on, complementary feeding was introduced to cover energy and nutrient needs. The quality of this complementary feeding becomes more relevant because of the reduction in exclusive breastfeeding time in developed countries [3]. In general, it is considered that babies are prepared to eat solid foods from 6 months of age. The diet that complements breast milk after this age should consist of a combination of pureed or strained fruits (banana, pears, applesauce, peaches, avocado), pureed or strained vegetables (well-cooked carrots, squash, sweet potato), pureed meat (chicken, pork, beef), pureed legumes (black beans, chickpeas, edamame, fava beans, black-eyed peas, lentils, kidney beans), iron-fortified cereal (oats, barley), and small amounts of unsweetened yogurt. From the 8th month, they should be also introduced to fish intake as a protein source, always in the form of puree. According to official data, a Spanish child consumes around 44 kilos of baby food per year, and it represents sales of more than 110 million euros per year [4]. While this type of food could only be purchased in pharmacies a few years ago, it is now distributed in supermarkets and other establishments, which facilitates the population’s access to this type of food and has allowed the introduction of store brands and lower-cost products into the market. The consumption of ready-to-eat baby food in Spain is concentrated in urban areas, especially in large cities, where the current lifestyle, with many families in which both parents work full time away from home, has introduced new eating habits in babies, as parents have less time to prepare homemade food [4]. Nutritional surveys in the under-two population are limited to non-official studies or focused on types of nutrients rather than types of food. Thus, for children aged 8–12 months, it has been estimated that vegetable purees account for 26% of the daily ration and fruit purees for 18%. The average daily ration was estimated at 260 and 182 g, respectively [5]. The manufacture of this type of food is strictly regulated by both national and European laws [6]. While it is assumed that the commercial baby diet is carefully formulated to ensure the supply of all the necessary nutrients to the infant, it is equally important to ensure the absence of high levels of non-essential or toxic elements that may come from the raw materials employed in the formulation of these foods, or well derived from the deficiencies in the manufacturing or production processes, with a special focus on low-cost products. Thus, depending on the type of food, the upper and lower limits of essential chemical elements such as zinc, copper, or selenium are legislated [6]. This regulation does not imply that some studies report nutritional imbalance in this segment of the population [7]. Similarly, the maximum levels of the most hazardous chemical elements, mainly heavy metals, are legislated at both the European and national levels [8], although there are many other elements for which there is no legislation.

In recent decades, living beings have been progressively exposed to naturally occurring substances that have remained alien to the earth’s surface environment. It can be explained by the discovery or rediscovery of interesting physicochemical properties of a wide range of natural elements commonly used for technological development [9]. As a consequence, living beings are now increasingly exposed to an unprecedented variety of toxic or potentially toxic elements that are mobilized from places in remote locations where they are located (mines) and enter in the environment as a result of human activities, ranging from coal-fired power plants to waste incinerators, to the manufacturing industry of high-tech electronic devices [10,11,12]. These “emerging elements” are mainly rare earth elements (REEs) and other minor elements (MEs), highly coveted due to their peculiar properties (electronic configuration) that make them very useful (or almost indispensable) for the manufacturing of all kinds of today’s technological devices [9]. The whole range of the toxicological effects of many of these elements are unknown to date, but, based on the few evidences available, many of these REEs and MEs have been included among the emerging occupational and environmental health risks by several international organizations [13]. There are currently very few studies associating exposure to these elements with adverse health effects. Associations with acute ischemic stroke [14] or with blood parameters such as anemia [15] have been observed, although the evidence is still insufficient. However, studies emphasize sensitive segments of the population, including children [12,16]. In addition, the number of studies proposing these chemicals as health risk factors is increasing [12,14,15], although this is true even when it refers to elements that are essential for life but that can be toxic when exposure to them is excessively high [17]. This growing exposure is a cause for concern about its adverse effects on health, especially in children. Thus, the Agency for Toxic Substances and Disease Registry (ATSDR) has included the most toxic chemical elements in its biannual list of priority pollutants [18].

It has been well established that the main route of exposure of the general population to these contaminants is food, and there are many studies in the literature that report high levels of elements either in foods used as raw materials or in processed foods [19,20,21,22]. Nutritional assessments have showed that some foods can be a major source of contamination for humans [23] and animals [24]. The presence of “emerging elements” in food has been less studied. Although there are some studies available to date [25,26,27], no official food safety organization has established limits to their presence in food or recommendations regarding the maximum exposure to them. However, some independent researchers have proposed some reference values for them considered as a group [25].

In order to increase the knowledge of infant exposure to chemical elements, the present study aims (i) to determine the content of 38 elements in 159 samples of ready-to-eat baby food sold in Spain. The selected list of elements includes both essential and non-essential/toxic elements and represents the first study reporting the levels of a wide range of hi-tech-related elements that are currently considered as emerging environmental pollutants in baby food; and, despite the limitations, inferences and assumptions that this type of analysis has, due to the specificity of the population and the lack of rigorous information for some of the chemicals considered, (ii) to estimate the dietary intake and risk assessment of these elements in infants ranging between 6 and 12 months of age, considering two scenarios: (a) infants consuming name-brand products and (b) infants consuming low-cost products (generic or store brands).

## 2. Materials and Methods

### 2.1. Sampling

The samples were selected based on the sales volume in different establishments, choosing the most common brands due to their presence in the retail stores. The sample size was determined by the range of brands, which was expanded by including different varieties, maintaining parity between the two types of brands. A total of 102 name brands and 57 store brands were purchased. The term “name brands” referred to recognized and established brands that carry a specific name, whereas “store brands” referred to private label products, generally cheaper. Each brand was acquired from specialized stores and supermarkets located in the island of Gran Canaria (Spain). All samples had an expiration date exceeding 6 months from the date of purchase. The 159 samples were distributed as follows: 40 fruit purees, 39 chicken purees, 40 fish purees, and 40 beef purees. All samples underwent national and/or international distribution; however, none of the food products were locally manufactured. The samples were stored in commercial packaging, without being removed, in a dark and dry environment at room temperature until analysis. The sampling method employed was similar to the one previously utilized by our group [23,24,28]. Sampling was made in October–December 2022.

### 2.2. Standards and Elements

A total of 38 chemical elements were analyzed, including essential elements, elements contained in the priority list of the ATSDR, and REEs and other MEs. The complete list of elements was as follows: iron, zinc, copper, selenium, manganese, molybdenum, and chromium (essential elements); silver, arsenic, aluminum, barium, beryllium, cadmium, mercury, nickel, lead, antimony, strontium, thallium, uranium, and vanadium (ATSDR priority list); and lanthanum, cerium, praseodymium, neodymium, promethium, samarium, europium, gadolinium, terbium, dysprosium, holmium, erbium, thulium, ytterbium, lutetium, scandium, and yttrium (REEs and MEs).

The internal standard solution included scandium, germanium, rhodium, and iridium (20 mg/mL each). Elements of standard purity (5% HNO_3_, 100 mg/L) were purchased from CPA Chem (Stara Zagora, Bulgaria). Two standard curves (range = 0.005–100 ng/mL) were made: one containing the essential trace elements and the main heavy metals (CPA Chem Catalog number E5B8·K1.5N.L1, 21 elements), and (b) the other contained the REEs and other elements used in electronic devices (CPA Chem). Quality of analyses and quality controls have also been previously published [23,24].

### 2.3. Sample Preparation and Analytical Procedure

The samples were thoroughly mixed and manually homogenized. To each sample, the following components were added: 50 µL of the internal standard, 2.5 mL of nitric acid (65%), and 7.5 mL of Milli-Q water. A total of 500 mg were acid digested in a microwave digester (Ethos Up, Milestone SRL, Sorisole, Italy) as follows: Step 1: power (W), temperature (C), and time (min) of 1800, 100, and 5, respectively; Step 2: 1800, 150, and 5; Step 3: 1800, 200, and 8; Step 4: 1800, 200, and 7, as previously reported [23,24]. The digested samples were transferred quantitatively into conical bottom polypropylene tubes and diluted up to 15 mL with Milli-Q water. From each digestion vessel, three samples were taken to obtain triplicate measurements for each sample. Additionally, an analytical batch included a reagent blank prepared similarly to the samples, which was included every 14 samples.

An Agilent 7900 ICP-MS instrument (Agilent Technologies, Tokyo, Japan) was utilized for all measurements. The data acquisition and processing were performed using Agilent MassHunter Data Analysis software (version 4.2, Agilent Technologies, Palo Alto, CA, USA). Prior to the analysis of samples, the entire procedure underwent in-house validation to ensure its accuracy and reliability [23,24]. Recoveries obtained ranged from 87 to 118% for toxic and essential elements. Linear calibration curves were found for all elements (regression coefficients ≥ 0.998). The limit of quantification (LOQ) for the method was determined by quantifying twenty replicates of blanks using 0.130 μL of alkaline solution. The LOQs were calculated as the concentration of the element that generated a signal three times higher than the average signal of the blanks. The accuracy and precision of this method was assessed using fortified alkaline solution (0.05, 0.5, and 5 ng/mL) in substitution of sample. The calculated relative standard deviations were lower than 8%, except for some few elements (Cu, Ni, Se, Ba, Zn, Sm), as it raised to 15–16% at the lowest level of fortification. The precision improved at the highest level of concentration, as it was lower than 5% for all elements.

### 2.4. Estimation of Dietary Intake and Nutritional and Health Risk Assessment

For the estimation of the intake of chemical elements, the total consumption of ready-to-eat purees for babies was taken into account. This value of consumption (g/day) [29] was multiplied by the median values of each element (ng/g weight). The total consumption of each element (ng/kg body weight/day) was calculated. Both average consumers (those in the 50th percentile) and high consumers (those in the 97.5th percentile (P97.5)) were considered.

For the estimation of the risk–benefit ratio, the values of Estimated Daily Intake (EDI) of elements for each scenario (average and high consumers) were compared with the reference values. As Dietary Reference Values (in the case of the essential elements, DRVs), the Population Reference Intake (PRI) values, as reported by the European Food Safety Authority (EFSA), were used [30]. In cases where the EFSA did not provide the PRI, the Adequate Intake (AI) was used as the reference value. AI represented the average daily nutrient level consumed by a typical healthy population and was assumed to be sufficient for their nutritional requirements. Additionally, for estimates of essential elements that exceeded the PRI or AI, the Tolerable Upper Intake Level (UL) was taken into consideration. The UL represented the maximum level of chronic nutrient intake from all sources that was unlikely to cause adverse health effects in humans [31]. The non-carcinogenic Toxic Reference Values (TRVs) used in this study were based on the Tolerable Daily Intake (TDI) values provided by the US Environmental Protection Agency [32]. No official TRV was established either for the REEs or the other MEs included in this research. However, some authors proposed a daily allowable intake of 61 µg/kg body weight for these elements [25,27]. We used this value as the TRV for the sum of REEs in our study.

The estimated short-term intake (ESTI), as the acute health risk, was calculated as follows [33]:

ESTI = HRE × K

where HRE represents the highest residue level found for each element in the analyzed series, and K is the recommended amount of food per kilo and day. ESTI is measured in ng of element per kilogram of body mass per day.

The acute hazard index (aHI), as the ratio between the exposure to a single dose of a toxic substance and the acute reference dose of toxicity for it, was calculated as follows [24,28]:

$$\mathrm{aHI}=\frac{\mathrm{ESTI}}{\mathrm{ARfD}}$$

where ARfD represents the Acute Reference Dose, defined as an estimation of the amount of the maximum amount of a substance in food (or drinking water) expressed on a body mass basis, which can be ingested in a period of 24 h or less without appreciable health risks to the consumer [32].

We also calculated risk quotient (RQ), defined as the ratio of a point estimate of exposure and a point estimate of effects:

$$\mathrm{RQ}=\frac{\mathrm{Exposure}}{\mathrm{Toxicity}}$$

in terms of percentage of the tolerable daily intake or provisional tolerable weekly intake.

### 2.5. Statistical Analysis

Descriptive analyses were performed for all variables, including calculations of the mean, standard deviation, median, range, and proportions. For values below the LOQ, a random number between 0 and the LOQ was assigned [23,24,34]. Due to the non-normal distribution of most of the data series, non-parametric tests were utilized in the analysis. PASW Statistics v 19.0 (SPSS Inc., Chicago, IL, USA) was employed to manage the study database and conduct statistical analyses. A significance level of <0.05 (two-tailed) was considered statistically significant.

## 3. Results and Discussion

### 3.1. Content of Chemical Elements in Ready-to-Eat Purees for Babies

Concentration levels of essential elements and chemical elements included in the ATSDR’s list of priority pollutants are included in Table 1 and Table 2.

As expected, all essential elements were detected in 100% of samples. Iron was the chemical element present at the highest concentration in fruit purees, whereas zinc was the chemical element present at the highest concentration in chicken and beef purees, regardless of the brand. For the fish purees, iron had the highest concentration for name brands and zinc for store brands (Table 1). This finding was consistent with other nutritional analyses that reported higher iron and zinc intakes among children who consumed this kind of formulae [35,36]. We observed significant differences in molybdenum, chromium, zinc, and selenium concentrations between the different types of brands. In particular, the store brands had higher levels of these chemical elements (Table 1). Fruit purees had higher concentrations of molybdenum (*p* = 0.025) and chromium (*p* = 0.043), whereas no significant differences were observed for any essential element in the case of beef purees. This profile was observed in similar studies on pet foods, in which store brands showed higher concentrations of essential elements (iron and copper) [24].

A total of 12 of 15 (80.0%) chemical elements considered in this study and included in the ATSDR’s list of priority pollutants showed a detection frequency of 100% (Table 2). Beryllium was the chemical element least frequently detected, irrespective of brand type. Aluminum and strontium were the chemical elements showing the highest levels. In the case of aluminum, its levels were significantly higher among the name brands (*p* = 0.031). Fish purees showed the highest concentrations of mercury (28.1 ng/g), being significantly higher among name brands (*p* = 0.002). It is well known that fish is an important source of mercury, so the present result is in agreement with the literature [37]. Given that the World Health Organization recommends reducing the consumption of certain fish species in children, it may be appropriate to extend this recommendation to ready-to-eat fish purees, especially taking into account that data have demonstrated that even low levels of exposure of mercury are still an important health concern for children [38]. The fact that it was the name brands that had higher mercury levels suggested a higher proportion of fish in them. Unfortunately, the labelling of these products did not provide sufficient information to test this hypothesis. In contrast, the highest level of arsenic was observed in store label fish purees (346.2 ng/g), being significantly higher than those observed in name brands (212.5 ng/g, *p* = 0.001; Table 2). Fish and rice are the main sources of arsenic exposure [39,40]. Compared to the food with the lowest concentration of arsenic (5.5 ng/g in store brands of fruit purees), 63 times more arsenic was observed in the fish puree (Table 2). Finally, store brands of chicken purees showed three times more nickel than name brands (142.1 vs. 46.9 ng/g, respectively; *p* = 0.006). As was the case for the essential elements, no significant differences in the concentrations of these chemical elements were observed between brands for the beef purees (Table 2). In general, these results were in line with those reported in the pet food study, where store brands showed higher levels of this group of contaminants [24].

Individual levels of REEs in each sample of name and store brands of ready-to-eat baby purees are detailed in Supplementary Tables S1 and S2, respectively. Following the analysis strategy of other authors [25,27], subsequent analyses were carried out considering the sum of these chemical elements. We observed that store brands showed higher levels of ∑REEs than name brands (Table 3), specifically for the chicken (12.1 vs. 6.2 ng/g, respectively; *p* = 0.031) and beef (16.1 vs. 10.1 ng/g, respectively; *p* = 0.008) purees. The presence of REEs and other MEs in food has been observed before [26], so children’s food should not be an exception. In general, REE concentrations in food are quite variable and low but have been reported in a wide range of foods, including fresh vegetables, rice, cereals, fresh aquatic products, fresh meats, and eggs [35,41]. To our knowledge, this is the first time that many of these chemical elements have been analyzed in ready-to-eat purees for babies.

### 3.2. Dietary Intake and Risk Assessment

We calculated the estimated daily intake (EDI) of the essential chemical elements, for which there is an adequate intake (AI) value [30]. For this purpose, we simulated two dietary scenarios depending on the type of food consumed by the children: (a) babies consuming only name brands and (b) babies consuming only store brands (Table 4). The models considered an average consumption of fruit purees of 174 g/day and an average consumption of protein from chicken, fish, and beef purees of 58 g/day, based on the latest available data [29]. With these two pieces of information, we then calculated the AI percentage to find out which essential chemical elements might be consuming more than necessary. As shown in Table 4, estimated intakes of manganese, molybdenum, and chromium were higher than AIs, especially in the case of store brands. The level of molybdenum, whose intake was 2.3 times the AI consumers of store brands, has to be highlighted. Chromium also showed more than twice the adequate intake (Table 4). Of these three substances, two (chromium and manganese) are included in the ATSDR list of priority toxic substances. Moreover, chromium and other heavy metals are classified as potent Group I carcinogens and cause various types of cancer in humans [42]. Previous studies in children have observed this over-ingestion of chromium associated with the consumption of rice [43], which is often included in baby food as a complementary ingredient to the usual protein sources.

**Figure 1:**
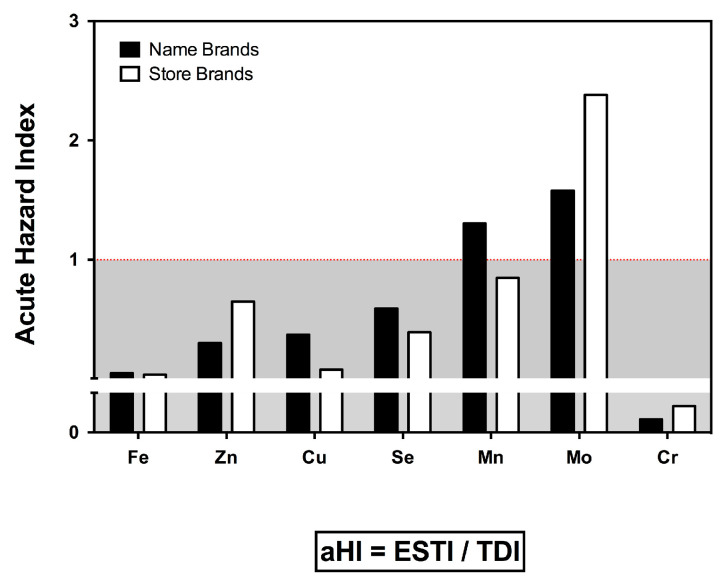
Bar plot indicating the acute hazard index (aHI) of essential chemical elements through the consumption of ready-to-eat purees for babies, in name and store brands.

In order to assess the risk linked to the consumption of chemical elements, we calculated the acute hazard index (aHI), as has been performed in previous studies [24]. The aHI defines the worst-case scenario in the series, considered as the risk of acute poisoning that an individual would be exposed to if the sample containing the highest concentration of the studied substance is ingested, considering all values below 1 as no risk of acute poisoning. Some considerations need to be made beforehand in order to interpret the following results correctly: first, the risk analyses are generic and should be interpreted with caution, as we are extrapolating them to a specific population of children aged 6 to 12 months; second, while it is true that clearer conclusions can be drawn for certain chemical elements (such as essential elements or heavy metals), this may not be possible for others (REEs and other MEs) due to insufficient studies that adequately assess the risk; and, finally, given these significant limitations, the following results should be considered as indicators particularly useful for future studies aiming to establish risk levels in this population segment and for all elements. Thus, while it is true that this index depends on the highest value in the series and is therefore a point measure of the sample, we observed aHI values above one for manganese and molybdenum (Figure 1). The danger of molybdenum is linked to the balance with copper [44], which makes it difficult to assess the significance of this finding, especially when the dietary intake of copper is almost 100% of the recommended daily intake (Table 4). Manganese, on the other hand, is detrimental to children’s physical and behavioral health [45,46]. However, the observed aHI is difficult to extrapolate, as the amount of manganese in food is strongly influenced by environmental and contamination conditions inherent to different foods [47,48].

**Figure 2:**
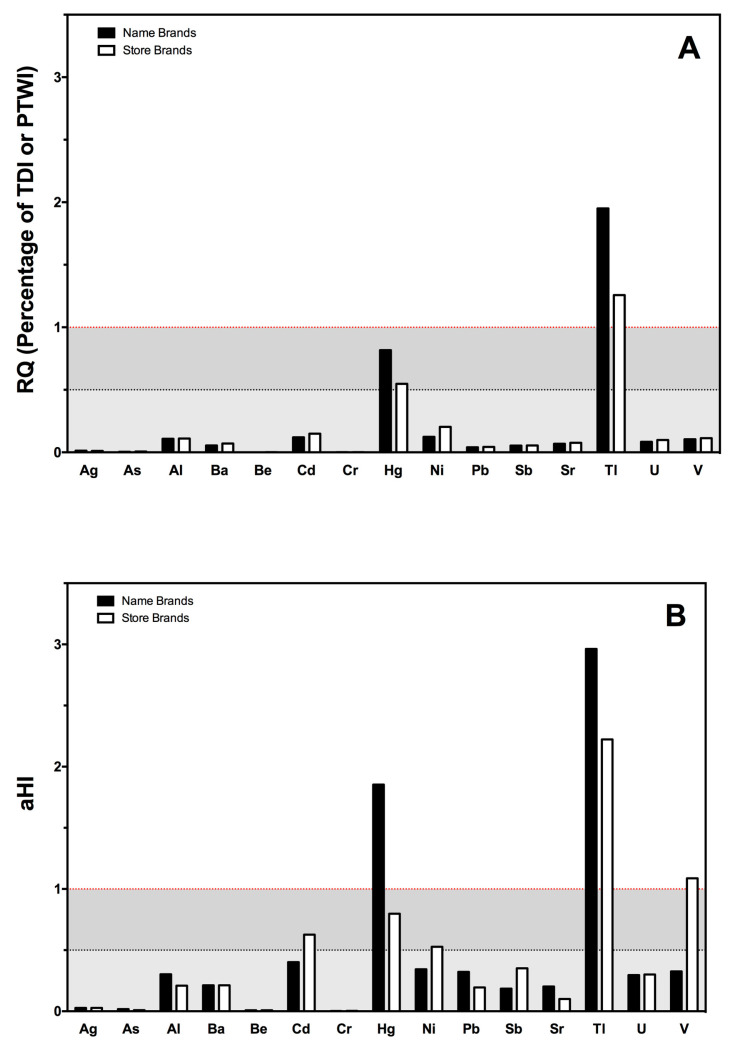
Bar plot indicating the Risk Quotients (RQ) (panel **A**) and aHI (panel **B**) of toxic chemical elements through the consumption of ready-to-eat purees for babies, in name and store brands. Horizontal lines indicate 100% and 50% of each index.

According to the Environmental Protection Agency (EPA) of the United States, Risk Quotients (RQs) are calculated by dividing a point estimate of exposure by a point estimate of effects. This ratio is a simple, screening-level estimate that identifies high- or low-risk situations. To estimate the risk associated with the daily ingestion of toxic chemicals, we calculated RQ in terms of percentage of the tolerable daily intake (TDI) or provisional tolerable weekly intake. Values below 1 are considered as no risk. As shown in Figure 2A, only thallium showed an RQ above 1. It is considered a highly toxic element, and, once in the body, it is absorbed in the gastrointestinal tract, widely distributed, and stored. Thallium concentrations have been detected in different types of foods [49]; to our knowledge, this is the first time it has been detected in children’s meals. Although interesting, the result should be taken with caution, as the indices used have limitations with regard to their accuracy and precision [50]. Therefore, to complete this information, we estimated the aHI of these potentially toxic chemicals (Figure 2B). Name brands showed high aHI values for mercury (almost 2×) and thallium (almost 3×). These are two highly toxic elements with very harmful effects on children [18] and should, therefore, be taken into account by Food Control Agencies. Although their levels are legislated—mercury in particular—it seems that some of these foods reach the final consumer, with the consequences that this may have.

**Figure 3:**
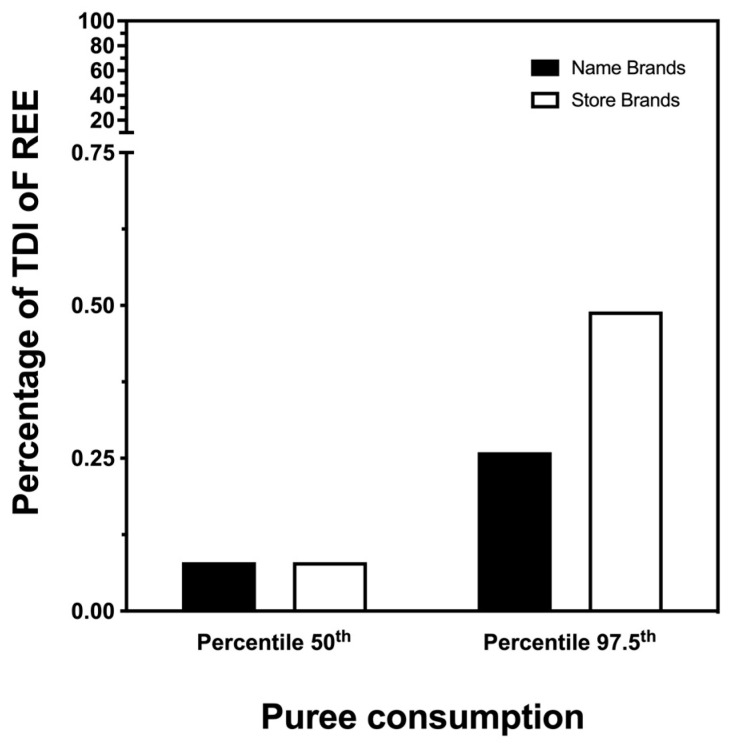
Bar plot indicating the percentages of tolerable daily intake (TDI) or provisional tolerable weekly intake of rare earth elements through the consumption of ready-to-eat purees for babies, in two consumptions models: children who were at the average consumption of the foods considered and children who were at the 97.5th percentile of consumption.

Finally, we wanted to assess the risk associated with exposure to REEs through these foods in the pediatric population. To do this, we took these chemical elements as a sum. As no maximum residue limits or maximum tolerable intakes had been established, we used the maximum exposure values proposed by other authors [25,27]. Furthermore, we conducted the analysis in two different exposure scenarios: children who were at the average consumption of the foods considered and children who were at the 97.5th percentile of consumption (Figure 3). In the first scenario, no risk associated with the intake of REEs was observed for either type of brand. In the second scenario, the maximum tolerable intake was reached, according to data reported by other authors [25,27]. These results are in line with those reported by other authors who observed low levels of REEs in different foods [23,41]. However, as no details of the toxicity or mechanism of action of most of these substances are known, and considering that they are capable of exerting adverse health effects even at low doses [49,51], this result should not be considered as safe in any case [52].

## 4. Conclusions

The present study showed that the baby foods analyzed showed the presence of chemical elements which, in some cases, were toxic, despite existing legislation. Fish purees showed the highest concentrations of mercury and arsenic. The risk analyses showed that the levels of essential chemical elements such as manganese, molybdenum, or chromium exceed the percentage of AI, being higher in the case of store brands. Both molybdenum and manganese had an aHI above 1. Among the chemical elements considered toxic by international agencies, we observed a risky consumption of thallium and mercury, being higher among name brands. The risk associated with the consumption of REEs was low, although biomonitoring studies are needed in at-risk populations to assess the consequences that these substances may be having on the health of individuals, given the lack of knowledge about these substances and the annual increase in exposure levels. This is the first time that these chemical elements have been measured in infant foods of this type. The results suggest that food quality controls should be maximized and that strategies should be implemented to establish maximum intake limits on a greater number of chemical elements considered by the scientific community as hazardous to health, including REEs and MEs.

## Figures and Tables

**Table 1:** Concentration of essential elements in ready-to-eat purees for babies in major name brands and store brands. The results are presented in ng/g fresh product.

	**FRUIT PUREES**						
	**Name Brands (*n* = 24)**			**Store Brands (*n* = 16)**			
	**Median**	**Range**	**Freq**	**Median**	**Range**	**Freq**	***p***
Fe	2791.3	754.7–11,995.5	100	3593.3	2030.9–9187.9	100	n.s.
Zn	1233.0	311.3–2012.8	100	1832.6	102.2–3685.9	100	n.s.
Cu	1295.3	197.9–4622.7	100	1129.7	676.1–1366.7	100	n.s.
Se	17.4	9.4–43.7	100	23.0	12.2–36.1	100	n.s.
Mn	1408.3	451.1–7797.4	100	1720.6	605.9–4771.4	100	n.s.
Mo	44.2	11.7–246.6	100	74.1	58.3–436.7	100	0.025
Cr	31.6	2.0–112.4	100	51.4	15.6–188.2	100	0.043
	**CHICKEN PUREES**						
	**Name Brands (*n* = 28)**			**Store Brands (*n* = 15)**			
	**Median**	**Range**	**Freq**	**Median**	**Range**	**Freq**	***p***
Fe	6146.1	2899.7–18,641.2	100	6961.4	4361.3–15,365.8	100	n.s.
Zn	6574.9	4393.7–14,788.6	100	9331.9	7438.3–15,740.1	100	0.022
Cu	622.7	379.6–1977.4	100	1004.7	495.2–2770.7	100	n.s.
Se	55.9	28.2–263.3	100	81.4	46.3–159.4	100	n.s.
Mn	1281.6	497.4–3451.9	100	1996.6	948.5–3201.3	100	n.s.
Mo	92.7	25.2–449.5	100	133.7	65.1–555.3	100	n.s.
Cr	46.2	17.1–80.0	100	59.8	25.4–354.4	100	n.s.
	**FISH PUREES**						
	**Name Brands (*n* = 28)**			**Store Brands (*n* = 12)**			
	**Median**	**Range**	**Freq**	**Median**	**Range**	**Freq**	***p***
Fe	4751.3	263.4–8395.9	100	4710.9	3151.2–6270.8	100	n.s.
Zn	4199.6	2232.6–6811.2	100	6105.4	5399.6–18,722.1	100	0.001
Cu	872.3	7.5–2815.1	100	886.8	882.3–945.3	100	n.s.
Se	101.1	6.1–333.9	100	126.2	100.3–146.1	100	0.004
Mn	1406.9	98.7–4314.1	100	1773.5	1241.8–2305.2	100	n.s.
Mo	89.7	10.9–368.6	100	158.7	98.4–376.2	100	n.s.
Cr	28.7	7.8–130.9	100	35.6	10.1–155.4	100	n.s.
	**BEEF PUREES**						
	**Name Brands (*n* = 26)**			**Store Brands (*n* = 14)**			
	**Median**	**Range**	**Freq**	**Median**	**Range**	**Freq**	***p***
Fe	9601.2	4603.6–16,668.8	100	9881.3	6733.5–14,023.1	100	n.s.
Zn	11,853.8	7160.5–26,752.1	100	12,339.7	8086.5–18,165.7	100	n.s.
Cu	1059.3	778.0–2729.9	100	1128.5	988.7–1460.5	100	n.s.
Se	40.8	25.7–51.1	100	40.7	28.7–58.9	100	n.s.
Mn	1602.4	753.4–4370.1	100	1439.1	975.5–1725.5	100	n.s.
Mo	139.8	31.2–434.4	100	225.1	48.1–374.5	100	n.s.
Cr	37.2	14.3–383.9	100	40.5	17.1–118.3	100	n.s.

n.s. means not significant.

**Table 2:** Concentration of elements in the ATSDR’s list of priority pollutants in ready-to-eat purees for babies in major name brands and store brands. The results are presented in ng/g fresh product.

	**FRUIT PUREES**						
	**Name Brands (*n* = 24)**			**Store Brands (*n* = 16)**			
	**Median**	**Range**	**Freq**	**Median**	**Range**	**Freq**	***p***
Ag	2.6	1.3–5.4	100	1.9	0.9–5.6	100	n.s.
As	5.7	1.1–11.5	100	5.5	1.8–10.6	100	n.s.
Al	4204.2	1844.3–9116.5	100	2124.9	1369.5–4902.0	100	0.031
Ba	297.3	77.1–1512.6	100	422.3	227.8–1387.9	100	n.s.
Be	0.0	<LOQ–0.6	18	0.0	<LOQ–0.5	14	n.s.
Cd	0.5	<LOQ–1.6	72	0.5	<LOQ–3.8	82	n.s.
Cr	31.6	1.9–112.5	92	51.4	15.7–188.2	100	0.036
Hg	18.0	10.4–42.7	100	12.1	9.2–17.6	100	n.s.
Ni	51.9	4.3–140.7	100	84.9	21.4–244.9	100	0.008
Pb	4.4	0.9–45.0	100	4.5	1.8–27.2	100	n.s.
Sb	0.8	0.4–3.0	100	0.7	0.4–6.2	100	n.s.
Sr	1206.2	225.8–4248.8	100	1425.4	502.4–1853.9	100	n.s.
Tl	5.9	2.7–8.7	100	3.6	2.9–6.7	100	n.s.
U	0.4	<LOQ–1.1	44	0.4	< LOQ–1.5	73	n.s.
V	3.3	0.6–11.7	100	3.8	3.4–34.6	100	n.s.
	**CHICKEN PUREES**						
	**Name Brands (*n* = 28)**			**Store Brands (*n* = 15)**			
	**Median**	**Range**	**Freq**	**Median**	**Range**	**Freq**	***p***
Ag	1.6	0.9–6.4	100	2.6	1.0–5.5	100	n.s.
As	9.6	2.3–79.9	100	25.06	2.8–74.7	100	n.s.
Al	2172.6	1395.9–4521.7	100	2673.3	1592.5–9077.3	100	n.s.
Ba	654.4	<LOQ–2105.1	85	612.2	172.9–3004.4	100	n.s.
Be	0.0	<LOQ–0.6	12	0.0	<LOQ–0.9	8	n.s.
Cd	11.9	<LOQ–50.1	75	15.8	5.3–151.7	100	n.s.
Cr	46.2	17.0–80.1	89	59.8	25.4–354.4	100	0.045
Hg	10.7	7.8–12.6	100	12.0	9.0–18.7	100	0.042
Ni	46.9	19.5–71.5	100	142.1	32.0–310.1	100	0.006
Pb	6.1	3.3–31.8	100	5.9	4.7–19.6	100	n.s.
Sb	0.8	0.4–1.5	100	1.1	0.9–1.7	100	n.s.
Sr	1550.5	917.8–2830.2	100	2065.7	898.4–3537.3	100	n.s.
Tl	2.9	1.7–3.6	100	1.9	1.5–2.1	100	n.s.
U	0.9	0.4–8.7	100	1.2	0.7–6.9	100	n.s.
V	6.0	2.5–17.0	100	4.8	2.7–6.9	100	n.s.
	**FISH PUREES**						
	**Name Brands (*n* = 28)**			**Store Brands (*n* = 12)**			
	**Median**	**Range**	**Freq**	**Median**	**Range**	**Freq**	***p***
Ag	1.1	0.6–3.8	100	1.9	1.3–2.5	100	n.s.
As	212.5	10.1–374.2	100	346.2	127.7–434.82	100	0.001
Al	3852.1	1430.5–7963.2	100	7987.3	3567.6–11,367.3	100	<0.001
Ba	640.8	0–1359.3	87	829.9	223.3–972.5	100	n.s.
Be	0.0	<LOQ–0.5	17	0.1	<LOQ–1.1	13	n.s.
Cd	15.5	<LOQ–55.0	67	16.7	2.1–17.8	100	n.s.
Cr	28.1	1.7–130.9	92	32.8	7.9–97.8	100	n.s.
Hg	28.1	1.7–58.9	100	12.2	9.5–17.8	100	0.002
Ni	53.6	13.1–202.5	100	48.3	21.09–198.2	100	n.s.
Pb	8.0	1.6–37.7	100	6.6	4.5–18.9	100	n.s.
Sb	0.5	0.1–1.3	100	0.4	0.3–0.8	100	n.s.
Sr	3312.5	42.9–7926.0	100	2715.1	456.7–8201.3	100	n.s.
Tl	1.3	<LOQ–2.9	89	1.3	0.2–2.3	100	n.s.
U	1.2	<LOQ–3.1	76	2.1	<LOQ–2.3	57	n.s.
V	5.2	1.2–10.3	100	5.2	4.5–5.8	100	n.s.
	**BEEF PUREES**						
	**Name Brands (*n* = 26)**			**Store Brands (*n* = 14)**			
	**Median**	**Range**	**Freq**	**Median**	**Range**	**Freq**	***p***
Ag	2.0	0.6–3.8	100	2.8	1.3–3.4	100	n.s.
As	7.1	1.7–52.9	100	6.6	2.9–12.9	100	n.s.
Al	3673.6	2908.2–38,860.4	100	4517.6	3606.3–7499.5	100	n.s.
Ba	866.4	245.2–1585.6	100	921.8	566.9–2214.9	100	n.s.
Be	0.0	<LOQ–0.8	36	0.0	<LOQ–0.3	27	n.s.
Cd	11.7	4.1–26.8	100	17.5	10.4–25.1	100	n.s.
Cr	37.2	4.7–198.7		40.5	17.1–118.3	100	n.s.
Hg	4.3	3.4–9.4	100	4.2	3.5–5.8	100	n.s.
Ni	88.4	19.1–257.8	100	69.4	40.1–96.2	100	n.s.
Pb	6.9	3.8–12.8	100	8.2	4.2–27.3	100	n.s.
Sb	0.7	0.4–2.8	100	1.1	0.8–3.5	100	n.s.
Sr	2394.5	624.8–4670.7	100	2487.5	932.2–3675.4	100	n.s.
Tl	2.3	<LOQ–5.8	91	2.9	0.3–3.8	100	n.s.
U	1.6	0.3–4.5	100	1.4	0.4–3.6	100	n.s.
V	5.1	2.6–11.3	100	4.8	3.1–7.9	100	n.s.

n.s. means not significant.

**Table 3:** Concentration of the sum of REE in ready-to-eat purees for babies in major name brands and store brands. The results are presented in ng/g fresh product.

	NAME BRANDS		STORE BRANDS		
	Median	Range	Median	Range	*p*
Fruit purees	7.3	1.9–29.1	5.9	3.9–60.7	n.s.
Chicken purees	6.2	3.2–16.1	12.1	4.2–18.9	0.031
Fish purees	9.6	2.8–17.6	8.0	6.6–9.4	n.s.
Beef purees	10.1	3.8–52.5	16.1	12.8–26.8	0.008

n.s. means not significant.

**Table 4:** Estimated daily intake of essential elements from the jarred ready-to-eat purees for babies 6–12 months. Assessment has been performed on the median values of measured concentrations in samples. The model considers an average consumption of 174 g/day of fruit puree and 58 g/day of protein-based purees (averaged data of chicken, fish, and beef jars). The model has been applied to two different scenarios: ^a^ babies consuming only name brands; and ^b^ babies consuming only store brands.

		CONSUMERS OF NAME BRANDS		CONSUMERS OF STORE BRANDS	
Essential Element	AI ^a^ (mg/Day)	EDI ^b^ (mg/Day)	% AI	EDI ^b^ (mg/Day)	% AI
Fe	8	0.88	11.02	1.04	13.02
Cu	0.3	0.27	91.59	0.25	84.99
Zn	5	0.65	13.04	0.86	17.12
Se	0.015	0.01	45.65	0.01	58.74
Mn	0.3	0.33	109.33	0.40	133.37
Mo	0.01	0.01	146.16	0.02	229.02
Cr	0.0055	0.01	139.50	0.01	210.26

## Data Availability

All data were included in the study (see Supplementary Materials).

## Supplementary Materials

The following supporting information can be downloaded at: https://www.mdpi.com/article/10.3390/nu15143251/s1, Table S1: Individual levels of REE in each sample of Name Brands of ready-to-eat baby purees. Results expressed in ng/g fresh product; Table S2: Individual levels of REE in each sample of Store Brands of ready-to-eat baby purees. Results expressed in ng/g fresh product.
