# Supplementary material for: Concentration of Essential, Toxic, and Rare Earth Elements in Ready-to-Eat Baby Purees from the Spanish Market
Source: Nutrients. 2023 Jul 22;15(14):3251. doi: 10.3390/nu15143251 (PMC10384816; doi:10.3390/nu15143251)
Supplement: Supplementary file 1 [file nutrients-15-03251-s001.zip › Supplementary Table S1.pdf]

Supplementary Table S1. Individual levels of REE in each sample of Name Brands of ready-to-eat baby purees. Results expressed in ng/g fresh product.

	La	Ce	Pr	Nd	Pm	Sm	Eu	Gd	Tb	Dy	Ho	Er	Tm	Yb	Lu	Sc	Y
**Fruit 01**	4.92	8.91	1.14	4.25	0.05	0.89	0.18	0.74	0.11	0.57	0.12	0.44	0.05	0.25	0.06	3.06	3.37
**Fruit 02**	0.52	0.79	0.14	0.37	0.23	0.14	0.09	0.16	0.08	0.15	0.07	0.20	0.06	0.09	0.07	1.04	0.47
**Fruit 03**	0.27	0.38	0.06	0.18	0.10	0.02	0.02	0.03	0.01	0.02	0.01	0.06	0.01	0.02	0.01	0.56	0.15
**Fruit 04**	1.08	2.01	0.25	0.93	0.16	0.28	0.05	0.20	0.05	0.19	0.03	0.21	0.01	0.09	0.02	0.95	0.96
**Fruit 05**	1.26	1.88	0.24	0.74	0.05	0.15	0.05	0.16	0.03	0.21	0.03	0.25	0.01	0.10	0.01	1.26	0.68
**Fruit 06**	1.99	3.50	0.45	1.88	0.15	0.35	0.09	0.36	0.04	0.23	0.06	0.33	0.03	0.09	0.02	1.43	1.63
**Fruit 07**	0.67	1.04	0.14	0.49	0.10	0.12	0.04	0.10	0.02	0.08	0.01	0.13	0.01	0.04	0.01	1.21	0.51
**Fruit 08**	0.68	1.48	0.16	0.63	0.02	0.15	0.03	0.15	0.02	0.11	0.01	0.15	0.01	0.05	0.01	1.29	0.75
**Fruit 09**	2.11	5.35	0.62	2.26	0.06	0.47	0.13	0.52	0.09	0.46	0.08	0.31	0.04	0.20	0.04	1.24	2.65
**Fruit 10**	2.11	3.12	0.34	1.51	0.07	0.27	0.08	0.31	0.04	0.28	0.05	0.23	0.02	0.13	0.02	1.09	1.89
**Fruit 11**	0.80	1.42	0.13	0.64	0.02	0.13	0.03	0.12	0.02	0.10	0.01	0.10	0.00	0.04	0.01	0.92	0.53
**Fruit 12**	2.17	4.62	0.55	2.16	0.10	0.43	0.08	0.41	0.05	0.18	0.03	0.14	0.01	0.06	0.02	1.21	0.91
**Fruit 13**	5.80	10.51	1.35	5.01	0.05	1.05	0.22	0.87	0.13	0.67	0.14	0.51	0.05	0.30	0.07	3.60	3.97
**Fruit 14**	0.61	0.93	0.17	0.43	0.27	0.16	0.10	0.19	0.09	0.17	0.08	0.24	0.06	0.10	0.08	1.23	0.55
**Fruit 15**	0.32	0.45	0.07	0.22	0.12	0.02	0.03	0.03	0.02	0.03	0.01	0.07	0.01	0.02	0.01	0.66	0.17
**Fruit 16**	1.27	2.37	0.30	1.10	0.19	0.33	0.06	0.24	0.05	0.22	0.04	0.25	0.01	0.10	0.02	1.12	1.13
**Fruit 17**	1.49	2.22	0.28	0.88	0.06	0.18	0.06	0.19	0.04	0.25	0.03	0.29	0.01	0.12	0.01	1.49	0.80
**Fruit 18**	2.34	4.13	0.53	2.21	0.18	0.41	0.10	0.42	0.05	0.27	0.07	0.39	0.03	0.11	0.02	1.69	1.92
**Fruit 19**	0.60	0.92	0.12	0.43	0.08	0.11	0.04	0.09	0.01	0.07	0.01	0.11	0.00	0.04	0.01	1.07	0.45
**Fruit 20**	0.60	1.31	0.14	0.55	0.02	0.13	0.02	0.13	0.02	0.10	0.01	0.13	0.01	0.04	0.01	1.14	0.66
**Fruit 21**	1.87	4.73	0.55	2.00	0.06	0.42	0.11	0.46	0.08	0.41	0.07	0.27	0.03	0.17	0.04	1.09	2.35
**Fruit 22**	1.87	2.76	0.30	1.33	0.06	0.24	0.07	0.27	0.04	0.25	0.04	0.20	0.02	0.12	0.01	0.96	1.67
**Fruit 23**	0.71	1.25	0.11	0.56	0.01	0.11	0.02	0.11	0.02	0.08	0.01	0.09	0.00	0.03	0.00	0.81	0.47
**Fruit 24**	1.92	4.08	0.49	1.91	0.09	0.38	0.07	0.36	0.04	0.16	0.03	0.12	0.00	0.05	0.01	1.07	0.80
**Chicken 01**	1.28	2.11	0.24	0.82	0.12	0.14	0.04	0.14	0.02	0.09	0.01	0.09	0.00	0.03	0.01	0.61	0.53
**Chicken 02**	2.59	5.88	0.61	2.32	0.09	0.65	0.10	0.37	0.06	0.25	0.04	0.16	0.01	0.04	0.01	1.31	1.67
**Chicken 03**	0.54	1.17	0.12	0.45	0.15	0.08	0.02	0.05	0.01	0.02	0.01	0.05	0.00	0.01	0.00	0.29	0.23
**Chicken 04**	1.89	2.99	0.36	1.34	0.05	0.30	0.07	0.24	0.03	0.12	0.04	0.09	0.01	0.05	0.01	0.70	0.98
**Chicken 05**	1.22	2.52	0.28	1.03	0.07	0.25	0.06	0.19	0.03	0.11	0.03	0.11	0.01	0.03	0.01	0.83	0.99
**Chicken 06**	1.84	3.52	0.40	1.42	0.07	0.25	0.10	0.26	0.04	0.23	0.04	0.15	0.02	0.08	0.01	1.07	1.27
**Chicken 07**	3.61	6.02	0.64	2.02	0.13	0.39	0.07	0.26	0.04	0.22	0.03	0.20	0.01	0.06	0.02	0.73	1.56
**Chicken 08**	0.67	1.26	0.14	0.47	0.08	0.08	0.01	0.09	0.01	0.05	0.01	0.09	0.00	0.01	0.00	0.56	0.49
**Chicken 09**	2.39	3.21	0.51	1.70	0.00	0.31	0.09	0.34	0.05	0.31	0.05	0.18	0.02	0.08	0.02	0.97	1.98
**Chicken 10**	0.74	1.32	0.16	0.45	0.08	0.10	0.03	0.09	0.01	0.06	0.01	0.07	0.00	0.03	0.02	0.36	0.59

**Chicken 11**	0.92	1.64	0.14	0.59	0.02	0.11	0.03	0.13	0.01	0.10	0.01	0.11	0.00	0.02	0.01	0.61	0.59
**Chicken 12**	0.90	1.76	0.18	0.67	0.08	0.13	0.05	0.15	0.02	0.11	0.01	0.13	0.00	0.04	0.01	0.46	0.90
**Chicken 13**	0.76	1.08	0.12	0.51	0.00	0.11	0.02	0.07	0.01	0.07	0.01	0.11	0.00	0.04	0.01	0.44	0.58
**Chicken 14**	0.58	0.99	0.07	0.43	0.10	0.05	0.02	0.05	0.01	0.04	0.01	0.10	0.00	0.03	0.01	0.46	0.27
**Chicken 15**	1.55	2.22	0.23	1.03	0.03	0.25	0.06	0.17	0.03	0.13	0.02	0.14	0.00	0.04	0.01	1.38	1.00
**Chicken 16**	1.51	2.48	0.28	0.97	0.14	0.16	0.05	0.16	0.03	0.10	0.01	0.10	0.00	0.03	0.01	0.71	0.62
**Chicken 17**	3.05	6.93	0.72	2.74	0.10	0.76	0.12	0.43	0.07	0.30	0.04	0.19	0.01	0.05	0.02	1.55	1.97
**Chicken 18**	0.63	1.38	0.14	0.53	0.17	0.10	0.02	0.06	0.01	0.02	0.01	0.06	0.00	0.01	0.00	0.34	0.27
**Chicken 19**	2.23	3.53	0.42	1.58	0.06	0.36	0.08	0.28	0.04	0.15	0.04	0.11	0.01	0.06	0.01	0.83	1.15
**Chicken 20**	1.44	2.97	0.33	1.21	0.08	0.29	0.07	0.22	0.03	0.13	0.03	0.13	0.01	0.04	0.01	0.97	1.16
**Chicken 21**	2.17	4.15	0.47	1.68	0.09	0.29	0.11	0.31	0.05	0.27	0.04	0.17	0.02	0.10	0.01	1.26	1.49
**Chicken 22**	4.26	7.10	0.76	2.38	0.15	0.46	0.08	0.31	0.04	0.25	0.03	0.24	0.01	0.06	0.02	0.86	1.84
**Chicken 23**	0.79	1.48	0.16	0.55	0.09	0.09	0.01	0.10	0.01	0.06	0.01	0.10	0.00	0.01	0.00	0.66	0.57
**Chicken 24**	2.82	3.79	0.60	2.01	0.00	0.36	0.11	0.40	0.06	0.37	0.06	0.22	0.02	0.09	0.02	1.14	2.34
**Fish 01**	2.51	5.51	0.54	2.27	0.00	0.57	0.08	0.39	0.06	0.25	0.06	0.26	0.02	0.12	0.02	2.26	1.62
**Fish 02**	3.73	7.02	0.39	1.69	0.00	0.32	0.07	0.31	0.04	0.25	0.05	0.19	0.01	0.08	0.02	2.28	1.18
**Fish 03**	0.89	1.32	0.12	0.44	0.00	0.05	0.04	0.10	0.02	0.10	0.02	0.10	0.00	0.03	0.01	0.39	0.66
**Fish 04**	1.38	2.09	0.20	0.81	0.00	0.18	0.05	0.14	0.02	0.15	0.02	0.19	0.01	0.06	0.01	1.04	0.67
**Fish 05**	1.58	3.58	0.37	1.28	0.00	0.28	0.07	0.23	0.04	0.19	0.03	0.21	0.01	0.08	0.02	1.72	1.09
**Fish 06**	1.56	3.25	0.31	1.40	0.00	0.35	0.07	0.26	0.04	0.21	0.04	0.22	0.01	0.10	0.01	1.72	1.06
**Fish 07**	0.74	1.28	0.11	0.55	0.00	0.13	0.03	0.10	0.01	0.10	0.03	0.15	0.00	0.05	0.01	0.49	0.50
**Fish 08**	1.64	3.02	0.34	1.37	0.00	0.28	0.05	0.30	0.04	0.25	0.04	0.20	0.02	0.11	0.02	1.46	1.84
**Fish 09**	1.88	3.85	0.43	1.67	0.00	0.32	0.08	0.27	0.04	0.26	0.05	0.26	0.01	0.09	0.02	1.72	1.28
**Fish 10**	1.65	2.94	0.33	1.48	0.00	0.27	0.06	0.24	0.04	0.19	0.05	0.24	0.01	0.09	0.02	1.02	1.56
**Fish 11**	1.06	2.24	0.27	1.09	0.00	0.20	0.07	0.22	0.05	0.23	0.04	0.21	0.01	0.07	0.02	1.70	0.90
**Fish 12**	0.47	0.96	0.12	0.51	0.00	0.10	0.01	0.08	0.02	0.09	0.02	0.14	0.00	0.03	0.01	0.51	0.48
**Fish 13**	0.72	1.33	0.14	0.54	0.00	0.13	0.02	0.14	0.02	0.06	0.02	0.13	0.00	0.06	0.01	0.58	0.59
**Fish 14**	1.14	1.88	0.21	0.74	0.00	0.17	0.03	0.17	0.02	0.16	0.04	0.23	0.01	0.08	0.01	0.85	0.73
**Fish 15**	2.22	4.87	0.48	2.00	0.00	0.50	0.07	0.35	0.05	0.22	0.05	0.23	0.02	0.11	0.02	2.00	1.43
**Fish 16**	3.30	6.21	0.35	1.49	0.00	0.28	0.06	0.27	0.03	0.22	0.04	0.17	0.01	0.07	0.01	2.02	1.05
**Fish 17**	0.78	1.17	0.11	0.39	0.00	0.04	0.03	0.09	0.02	0.09	0.02	0.09	0.00	0.02	0.01	0.34	0.58
**Fish 18**	1.22	1.85	0.18	0.72	0.00	0.16	0.04	0.12	0.01	0.13	0.02	0.17	0.00	0.05	0.01	0.92	0.59
**Fish 19**	1.40	3.16	0.32	1.14	0.00	0.25	0.06	0.20	0.03	0.16	0.03	0.19	0.01	0.07	0.02	1.52	0.96
**Fish 20**	1.38	2.87	0.27	1.23	0.00	0.31	0.06	0.23	0.03	0.18	0.03	0.19	0.01	0.09	0.01	1.52	0.94
**Fish 21**	0.66	1.14	0.10	0.49	0.00	0.12	0.03	0.09	0.01	0.08	0.03	0.14	0.00	0.05	0.00	0.43	0.44
**Fish 22**	1.45	2.67	0.30	1.22	0.00	0.25	0.05	0.26	0.04	0.22	0.03	0.17	0.02	0.09	0.02	1.29	1.63
**Fish 23**	1.66	3.41	0.38	1.48	0.00	0.28	0.07	0.24	0.03	0.23	0.05	0.23	0.01	0.08	0.01	1.52	1.13

**Fish 24**	1.46	2.60	0.29	1.31	0.00	0.23	0.05	0.21	0.04	0.17	0.04	0.22	0.01	0.08	0.02	0.90	1.38
**Fish 25**	0.93	1.98	0.23	0.96	0.00	0.18	0.06	0.20	0.04	0.20	0.04	0.19	0.01	0.06	0.02	1.50	0.80
**Fish 26**	0.41	0.85	0.10	0.45	0.00	0.08	0.01	0.07	0.02	0.08	0.02	0.12	0.00	0.03	0.00	0.45	0.42
**Fish 27**	0.64	1.17	0.12	0.47	0.00	0.12	0.02	0.12	0.02	0.06	0.02	0.12	0.00	0.06	0.01	0.52	0.52
**Fish 28**	1.01	1.66	0.18	0.66	0.00	0.15	0.03	0.15	0.02	0.14	0.03	0.20	0.01	0.07	0.01	0.75	0.64
**Beef 01**	9.57	20.74	2.34	8.84	0.00	1.55	0.11	1.21	0.13	0.57	0.09	0.27	0.02	0.13	0.02	4.80	2.20
**Beef 02**	1.51	2.35	0.22	0.80	0.00	0.21	0.06	0.19	0.03	0.10	0.03	0.14	0.00	0.05	0.01	0.87	0.70
**Beef 03**	2.93	6.71	0.75	3.04	0.00	0.50	0.08	0.48	0.07	0.38	0.08	0.26	0.03	0.21	0.02	1.99	1.79
**Beef 04**	2.64	5.16	0.52	2.08	0.00	0.40	0.10	0.39	0.07	0.35	0.07	0.22	0.02	0.13	0.02	2.35	1.57
**Beef 05**	1.79	3.18	0.31	1.25	0.00	0.27	0.08	0.25	0.04	0.21	0.03	0.14	0.01	0.09	0.01	1.41	1.18
**Beef 06**	1.63	2.96	0.31	1.16	0.00	0.31	0.07	0.25	0.04	0.22	0.04	0.18	0.02	0.10	0.02	1.16	1.53
**Beef 07**	1.47	2.37	0.26	1.13	0.00	0.26	0.05	0.19	0.04	0.19	0.03	0.16	0.01	0.06	0.02	3.64	1.29
**Beef 08**	1.43	2.78	0.26	1.10	0.00	0.27	0.05	0.24	0.04	0.15	0.03	0.17	0.01	0.06	0.01	1.04	1.10
**Beef 09**	2.41	4.55	0.51	1.67	0.00	0.27	0.09	0.23	0.03	0.10	0.03	0.15	0.01	0.06	0.01	2.28	0.88
**Beef 10**	1.31	2.33	0.23	0.91	0.00	0.17	0.06	0.19	0.03	0.13	0.03	0.14	0.01	0.06	0.01	1.26	0.85
**Beef 11**	3.08	5.82	0.55	2.13	0.00	0.41	0.13	0.53	0.07	0.35	0.09	0.35	0.03	0.14	0.02	2.35	2.96
**Beef 12**	1.64	2.76	0.24	1.01	0.01	0.23	0.04	0.17	0.02	0.11	0.04	0.20	0.01	0.06	0.01	2.30	1.02
**Beef 13**	1.65	3.13	0.33	1.24	0.00	0.30	0.09	0.26	0.05	0.19	0.05	0.25	0.02	0.09	0.02	1.67	1.97
**Beef 14**	11.30	24.47	2.76	10.43	0.00	1.83	0.13	1.42	0.15	0.67	0.10	0.32	0.02	0.15	0.02	5.66	2.59
**Beef 15**	1.78	2.77	0.25	0.95	0.00	0.25	0.06	0.22	0.03	0.12	0.03	0.16	0.00	0.06	0.01	1.03	0.83
**Beef 16**	3.46	7.92	0.88	3.58	0.00	0.59	0.09	0.56	0.08	0.45	0.09	0.30	0.04	0.25	0.02	2.35	2.11
**Beef 17**	3.12	6.09	0.61	2.45	0.00	0.48	0.11	0.46	0.08	0.41	0.08	0.26	0.02	0.15	0.03	2.77	1.85
**Beef 18**	2.11	3.75	0.37	1.48	0.00	0.31	0.09	0.30	0.04	0.25	0.04	0.17	0.01	0.11	0.01	1.66	1.39
**Beef 19**	1.92	3.49	0.37	1.37	0.00	0.36	0.09	0.30	0.05	0.26	0.05	0.21	0.02	0.12	0.02	1.37	1.81
**Beef 20**	1.73	2.79	0.31	1.34	0.00	0.30	0.06	0.23	0.04	0.22	0.04	0.19	0.01	0.07	0.02	4.29	1.53
**Beef 21**	1.68	3.28	0.31	1.30	0.00	0.32	0.05	0.28	0.04	0.18	0.04	0.20	0.01	0.06	0.01	1.23	1.30
**Beef 22**	2.84	5.37	0.60	1.97	0.00	0.31	0.10	0.27	0.03	0.12	0.04	0.17	0.01	0.06	0.02	2.69	1.03
**Beef 23**	1.15	2.06	0.20	0.81	0.00	0.15	0.05	0.17	0.03	0.11	0.02	0.13	0.01	0.05	0.01	1.12	0.75
**Beef 24**	2.72	5.15	0.48	1.88	0.00	0.36	0.11	0.46	0.06	0.31	0.08	0.31	0.02	0.12	0.02	2.08	2.62
**Beef 25**	1.45	2.44	0.21	0.89	0.01	0.21	0.04	0.15	0.02	0.10	0.03	0.18	0.01	0.05	0.01	2.04	0.90
**Beef 26**	1.46	2.77	0.29	1.09	0.00	0.27	0.08	0.23	0.04	0.17	0.04	0.22	0.01	0.08	0.01	1.48	1.74
