# Supplementary material for: Concentration of Essential, Toxic, and Rare Earth Elements in Ready-to-Eat Baby Purees from the Spanish Market
Source: Nutrients. 2023 Jul 22;15(14):3251. doi: 10.3390/nu15143251 (PMC10384816; doi:10.3390/nu15143251)
Supplement: Supplementary file 1 [file nutrients-15-03251-s001.zip › Supplementary Table S2.pdf]

Supplementary Table S2. Individual levels of REE in each sample of Store Brands of ready-to-eat baby purees. Results expressed in ng/g fresh product.

	La	Ce	Pr	Nd	Pm	Sm	Eu	Gd	Tb	Dy	Ho	Er	Tm	Yb	Lu	Sc	Y
**Fruit 01**	4.918	8.908	1.143	4.248	0.045	0.888	0.183	0.740	0.110	0.565	0.120	0.435	0.045	0.250	0.060	3.055	4.918
**Fruit 02**	0.518	0.788	0.140	0.368	0.233	0.138	0.085	0.163	0.078	0.145	0.068	0.200	0.055	0.088	0.070	1.043	0.518
**Fruit 03**	0.270	0.380	0.060	0.183	0.100	0.020	0.023	0.025	0.013	0.023	0.005	0.060	0.005	0.020	0.010	0.558	0.270
**Fruit 04**	1.078	2.010	0.253	0.930	0.158	0.283	0.050	0.200	0.045	0.185	0.033	0.210	0.010	0.085	0.018	0.945	1.078
**Fruit 05**	1.263	1.880	0.240	0.742	0.050	0.150	0.048	0.163	0.033	0.208	0.025	0.245	0.010	0.098	0.008	1.260	1.263
**Fruit 06**	1.985	3.503	0.453	1.875	0.153	0.345	0.085	0.355	0.040	0.230	0.060	0.333	0.028	0.090	0.018	1.430	1.985
**Fruit 07**	0.673	1.043	0.138	0.485	0.095	0.120	0.040	0.100	0.015	0.083	0.010	0.125	0.005	0.040	0.010	1.213	0.673
**Fruit 08**	0.678	1.478	0.160	0.625	0.018	0.145	0.028	0.148	0.020	0.110	0.010	0.148	0.008	0.048	0.008	1.285	0.678
**Fruit 09**	2.113	5.350	0.618	2.260	0.063	0.473	0.125	0.515	0.093	0.460	0.080	0.305	0.035	0.195	0.043	1.235	2.113
**Fruit 10**	2.113	3.120	0.343	1.508	0.073	0.270	0.078	0.305	0.040	0.278	0.045	0.228	0.023	0.130	0.015	1.090	2.113
**Fruit 11**	0.798	1.418	0.125	0.635	0.015	0.125	0.025	0.123	0.018	0.095	0.013	0.098	0.003	0.035	0.005	0.920	0.798
**Fruit 12**	1.272	2.372	0.299	1.097	0.186	0.334	0.059	0.236	0.053	0.218	0.039	0.248	0.012	0.100	0.021	1.115	1.272
**Fruit 13**	1.490	2.218	0.283	0.876	0.059	0.177	0.057	0.192	0.039	0.245	0.030	0.289	0.012	0.116	0.009	1.487	1.490
**Fruit 14**	2.342	4.134	0.535	2.213	0.181	0.407	0.100	0.419	0.047	0.271	0.071	0.393	0.033	0.106	0.021	1.687	2.342
**Fruit 15**	0.794	1.231	0.163	0.572	0.112	0.142	0.047	0.118	0.018	0.098	0.012	0.148	0.006	0.047	0.012	1.431	0.794
**Fruit 16**	0.821	1.432	0.173	0.770	0.092	0.341	0.146	0.118	0.112	0.298	0.045	0.132	0.232	0.000	0.00	1.112	0.421
**Chicken 01**	1.283	2.105	0.238	0.818	0.115	0.138	0.040	0.138	0.023	0.088	0.008	0.085	0.000	0.025	0.008	0.605	1.283
**Chicken 02**	2.588	5.875	0.613	2.318	0.085	0.648	0.098	0.365	0.060	0.253	0.038	0.163	0.010	0.040	0.013	1.310	2.588
**Chicken 03**	0.538	1.168	0.120	0.448	0.145	0.083	0.015	0.048	0.005	0.020	0.008	0.050	0.000	0.005	0.003	0.290	0.538
**Chicken 04**	1.893	2.993	0.358	1.335	0.050	0.303	0.068	0.235	0.030	0.123	0.038	0.090	0.008	0.053	0.005	0.703	1.893
**Chicken 05**	1.218	2.515	0.278	1.025	0.070	0.245	0.058	0.185	0.028	0.113	0.025	0.113	0.008	0.033	0.010	0.825	1.218
**Chicken 06**	1.843	3.518	0.395	1.420	0.073	0.245	0.095	0.263	0.043	0.225	0.035	0.145	0.015	0.083	0.010	1.068	1.843
**Chicken 07**	3.608	6.018	0.640	2.015	0.128	0.390	0.068	0.263	0.038	0.215	0.025	0.203	0.005	0.055	0.018	0.728	3.608
**Chicken 08**	0.668	1.258	0.138	0.465	0.078	0.075	0.010	0.085	0.008	0.050	0.010	0.085	0.000	0.008	0.003	0.558	0.668
**Chicken 09**	2.388	3.210	0.508	1.700	0.000	0.308	0.090	0.335	0.050	0.310	0.050	0.183	0.015	0.078	0.020	0.970	2.388
**Chicken 10**	0.742	1.318	0.155	0.448	0.078	0.095	0.030	0.085	0.005	0.063	0.010	0.073	0.003	0.025	0.015	0.363	0.742
**Chicken 11**	0.915	1.643	0.138	0.588	0.015	0.108	0.033	0.125	0.013	0.103	0.008	0.110	0.000	0.020	0.008	0.605	0.915
**Chicken 12**	0.903	1.760	0.180	0.668	0.078	0.133	0.050	0.150	0.018	0.110	0.013	0.125	0.003	0.035	0.008	0.460	0.903
**Chicken 13**	1.580	3.330	0.420	1.438	0.000	0.295	0.145	0.248	0.035	0.170	0.038	0.310	0.008	0.083	0.015	1.043	1.580
**Chicken 14**	0.473	0.805	0.058	0.305	0.000	0.038	0.015	0.030	0.008	0.053	0.010	0.070	0.000	0.008	0.003	0.753	0.473
**Chicken 15**	1.680	3.548	0.373	1.453	0.000	0.215	0.085	0.338	0.035	0.238	0.053	0.150	0.010	0.125	0.018	1.333	1.680
**Fish 01**	1.260	2.798	0.305	1.095	0.000	0.270	0.060	0.250	0.035	0.193	0.045	0.225	0.008	0.073	0.010	1.720	1.090
**Fish 02**	0.730	1.305	0.123	0.470	0.000	0.108	0.020	0.103	0.015	0.110	0.020	0.150	0.000	0.035	0.005	2.908	0.495
**Fish 03**	0.885	1.323	0.123	0.443	0.000	0.050	0.038	0.100	0.020	0.098	0.020	0.100	0.000	0.028	0.008	0.388	0.658

**Fish 04**	1.383	2.088	0.198	0.813	0.000	0.183	0.045	0.140	0.015	0.148	0.018	0.193	0.005	0.055	0.010	1.043	0.668
**Fish 05**	0.718	1.325	0.140	0.535	0.000	0.133	0.018	0.135	0.018	0.063	0.023	0.130	0.003	0.063	0.010	0.583	1.088
**Fish 06**	1.140	1.875	0.205	0.742	0.000	0.170	0.033	0.168	0.023	0.155	0.035	0.225	0.008	0.078	0.013	0.848	1.063
**Fish 07**	1.487	3.302	0.360	1.292	0.000	0.319	0.071	0.295	0.041	0.228	0.053	0.266	0.009	0.086	0.012	2.030	0.500
**Fish 08**	0.861	1.540	0.145	0.555	0.000	0.127	0.024	0.122	0.018	0.130	0.024	0.177	0.000	0.041	0.006	3.431	1.840
**Fish 09**	1.044	1.561	0.145	0.523	0.000	0.059	0.045	0.118	0.024	0.116	0.024	0.118	0.000	0.033	0.009	0.458	1.275
**Fish 10**	1.632	2.464	0.234	0.959	0.000	0.216	0.053	0.165	0.018	0.175	0.021	0.228	0.006	0.065	0.012	1.231	1.558
**Fish 11**	0.847	1.564	0.165	0.631	0.000	0.157	0.021	0.159	0.021	0.074	0.027	0.153	0.004	0.074	0.012	0.688	0.900
**Fish 12**	1.345	2.213	0.242	0.876	0.000	0.201	0.039	0.198	0.027	0.183	0.041	0.266	0.009	0.092	0.015	1.001	0.475
**Beef 01**	0.458	1.583	0.000	0.333	0.098	0.290	0.050	0.193	0.053	0.253	0.008	0.073	0.013	3.223	1.418	0.458	1.583
**Beef 02**	0.338	1.260	0.000	0.203	0.063	0.210	0.030	0.145	0.033	0.205	0.010	0.070	0.013	2.230	1.153	0.338	1.260
**Beef 03**	0.983	3.778	0.000	0.763	0.113	0.765	0.103	0.455	0.093	0.300	0.020	0.113	0.028	3.490	2.505	0.983	3.778
**Beef 04**	0.390	1.363	0.000	0.295	0.123	0.285	0.050	0.225	0.048	0.280	0.010	0.093	0.010	2.183	1.630	0.390	1.363
**Beef 05**	0.540	1.868	0.000	0.393	0.116	0.342	0.059	0.228	0.063	0.299	0.009	0.086	0.015	3.803	1.178	0.540	1.868
**Beef 06**	0.399	1.487	0.000	0.240	0.074	0.248	0.035	0.171	0.039	0.242	0.012	0.083	0.015	2.631	1.533	0.399	1.487
**Beef 07**	1.160	4.458	0.000	0.900	0.133	0.903	0.122	0.537	0.110	0.354	0.024	0.133	0.033	4.118	1.293	1.160	4.458
**Beef 08**	0.460	1.608	0.000	0.348	0.145	0.336	0.059	0.266	0.057	0.330	0.012	0.110	0.012	2.576	1.100	0.460	1.608
**Beef 09**	0.405	1.401	0.000	0.295	0.087	0.257	0.044	0.171	0.047	0.224	0.007	0.065	0.012	2.852	1.255	0.405	1.401
**Beef 10**	0.299	1.115	0.000	0.180	0.056	0.186	0.027	0.128	0.029	0.181	0.009	0.062	0.012	1.973	1.020	0.299	1.115
**Beef 11**	0.870	3.343	0.000	0.675	0.100	0.677	0.091	0.403	0.082	0.265	0.018	0.100	0.025	3.088	2.217	0.870	3.343
**Beef 12**	0.345	1.206	0.000	0.261	0.109	0.252	0.044	0.199	0.042	0.248	0.009	0.082	0.009	1.932	1.442	0.345	1.206
**Beef 13**	0.333	1.235	0.000	0.303	0.090	0.255	0.048	0.190	0.048	0.248	0.015	0.093	0.015	1.673	1.965	0.333	1.235
**Beef 14**	0.220	0.700	0.000	0.113	0.045	0.125	0.020	0.095	0.028	0.135	0.005	0.033	0.008	1.430	0.760	0.220	0.700
